# Long lasting insecticidal mosquito nets (LLINs) ownership, use and coverage following mass distribution campaign in Lake Victoria basin, Western Kenya

**DOI:** 10.1186/s12889-021-11062-7

**Published:** 2021-06-02

**Authors:** Peter N. Ng’ang’a, Polycarp Aduogo, Clifford M. Mutero

**Affiliations:** 1 International Centre of Insect Physiology and Ecology (ICIPE), PO Box 30772, Nairobi, Kenya; 2 School of Public Health, Jomo Kenyatta University of Agriculture and Technology, PO Box 62000, Nairobi, Kenya; 3 School of Health Systems and Public Health, University of Pretoria, University of Pretoria Institute for Sustainable Malaria Control (UP ISMC),, Private Bag X363, Pretoria, 0001 South Africa

**Keywords:** LLINs, Coverage, Usage, Households, Malaria, Control, Western Kenya

## Abstract

**Background:**

Long-lasting insecticidal nets (LLINs) are the most widely used malaria prevention and control intervention in Africa. However, their effectiveness may vary depending on their local geographic coverage, ownership and use at household level. This study aimed at assessing LLINs ownership and use following mass distribution campaign in western Kenya.

**Methods:**

A cross-sectional study was conducted in November 2017. A total of 160 households were randomly selected from 16 villages. Structured questionnaires were used to collect data on households’ knowledge on malaria, LLINs ownership, utilization and their perceived benefits. Data was analyzed using IBM Statistical Package for Social Sciences (SPSS) version 21 for windows. Variables were presented as proportions and associations between variables tested using Pearson’s chi-square test.

**Results:**

Malaria was reported to be the most frequently occurring disease (87.5%) in the area. Children under 5 years of age were reported to be at higher risks of malaria infection (28.6%). Around 31% of the respondents reported to have at least one member of the household sick with malaria a week before the interview. Commonly cited signs and symptoms of malaria were; fever (24.1%), headache (17.7%), vomiting (14.5%) feeling cold (12.6%) and loss of appetite (10%). There were 382 reported LLINs among 753 occupants in the 160 households surveyed. The average LLIN ownership was 2.4 nets per household and 1.97 persons per LLIN. Among the surveyed households, 96.9% owned at least one LLIN and 64.1% owned at least one LLIN for every two people. Among those who owned LLINs, 98.1% reported using them the previous night. Ownership per household ranged from 0 to 6 with a mean of 2.39. More than three quarter of the nets were acquired through free mass distribution campaigns and 80% were acquired less than 6 months prior to the survey.

**Conclusion:**

Despite high net coverage and use, a number of households experienced malaria episodes in the study area. There is need to investigate the likelihood of outdoor malaria transmission and assess the physical integrity of the existing LLINs and their insecticidal effectiveness in protecting household members against malaria.

**Supplementary Information:**

The online version contains supplementary material available at 10.1186/s12889-021-11062-7.

## Background

Globally, there were an estimated 229 million malaria cases in 2019 from 87 malaria endemic countries. Malaria deaths reduced steadily from 736,000 in 2000 to 409,000 in 2019 [1]. Of the 87 countries that were malaria endemic in 2019, 29 accounted for 95% of malaria cases globally. WHO African region recorded an estimated 215 million cases in 2019, accounting for about 94% of all cases [1]. Sub-Saharan Africa is the most affected by the disease, with nineteen countries accounting for almost 85% of the global malaria burden by 2019 [2]. Kenya is one of the sub-Saharan African countries where malaria is most prevalent and the disease accounts for 30% of all hospital outpatient attendance [3]. Western Kenya around Lake Victoria and the coastal regions have the highest malaria transmission levels in the country. Transmission in these areas is intense throughout the year with entomological inoculation rates of between 30 and 100 infectious bites per person per year [4].

Global decline in malaria cases since 2000 has mainly been attributed to the use of insecticide-treated nets [ITNs], particularly LLINs, indoor residual spraying [IRS] and improved malaria case management [5]. An insecticide-treated net (ITN) is a net (usually a bed net), designed to block mosquitoes physically, that has been treated with safe, residual insecticide for the purpose of killing and repelling mosquitoes, which carry malaria [6]. A long-lasting insecticide-treated net (LLIN) is an ITN designed to remain effective for multiple years without retreatment.

Multiple studies in sub-Saharan Africa have demonstrated that ITNs are highly effective in reducing malaria morbidity and mortality when used properly and consistently [6, 7]. Thus, WHO has recommended delivery of free-of-charge highly subsidized LLINs to maximize their coverage rates in endemic areas. In 2018, about half of all people at risk of malaria in Africa were protected by an insecticide-treated net, compared to 29% in 2010 [8]. In a study of Malaria prevention in the Kenyan highlands, it was found that sleeping under an insecticide treated net reduced the risk of malaria infection by 63% [9].

Between 2004 and 2015, approximately 50.2 million ITNs were distributed in Kenya, of which almost 49 million were of the LLIN variety. The distribution was undertaken through the routine system that started in October 2004 and distributed 23.3 million nets. The other routine free mass ITNs campaigns were in 2006, 2011–12 and 2014–2015 during which 26.9 million nets were distributed [3]. In 2011, the policy changed to cover the entire at-risk population regardless of age and gender while in 2014, the third round of mass LLIN distribution was launched to boost their coverage and replace the old ones [10, 11]. Among the areas that were targeted for universal coverage of LLIN were the highland epidemic and the endemic coastal region and the Lake Victoria basin. These mass campaigns significantly increased overall ITN coverage and have resulted in a continuous decline in malaria transmission in the targeted sites [12]. Free mass net distributions have been shown to reduce disparities in LLIN ownership resulting in better equity in coverage than when clinic-based or social marketing strategies are used [13–16].

However, attaining universal coverage and proper use of LLINs remains challenging in sub-Saharan Africa. In 2015, 52.5% of targeted households in Kenya had access to an LLIN but only 47.6% of the population used the device on a regular nightly basis [2]. Whether LLINs are effectively used to prevent malaria depends on a complex set of factors [11, 17, 18]. In a study in western Kenyan highlands, it was shown that seasonal patterns of precipitation and vector density, along with education, were associated with LLIN use [19]. Sleeping arrangements, such as sleeping on the floor (as opposed to a bed), and availability of areas amenable to hanging nets have also been shown to be associated with LLIN use [20, 21]. Other factors known to affect ITNs/LLINs use in developing countries include bednet ownership, age of bednet owner, gender, shape of the nets, inconvenience caused by hanging of the net?, heat discomfort inside a bednet due to limited airflow, marital status of user, distance to nearest health service where nets can be obtained, accessibility to transport, household size, bednet density and occupation of the household head [17, 19, 22–24].

The 2013 WHO Roll Back Malaria Monitoring and Evaluation Reference Group recommended four main indicators for measuring ITN/LLIN availability and use [25]. Two of the indicators are calculated at the household level, and the other two at the individual (population) level. The two household level indicators are (i) the proportion of households that own at least one ITN/LLIN and (ii) the proportion of households that own at least one ITN/LLIN for 2 people. The two population-level indicators are (iii) the proportion of the population with access to an ITN/LLIN within the household and (iv) the proportion of the population that used an ITN/LLIN the previous night [26].

While the ultimate goal of LLINs use is to protect the population against infected mosquitoes, their use needs to be understood in the context of coverage, availability, ownership and access indicators. The objective of this study was to assess LLINs coverage, ownership, utilization and access in a rural Kenyan community.

## Methods

### Study area

The study was conducted in Nyabondo plateau, an area located in Nyakach Sub-county of Kisumu County. It is located about 30 km North-East of Lake Victoria and lies between an altitude of 1520 m and 1658 m above sea level, and 0° 23′ 0 S and 34° 58′ 60 E. The area is host to an estimated 34,000 people with a high population density of nearly 460 persons per square kilometer (km) [27]. The community largely depends on brick making as the main economic activity with small scale mixed farming activities such as crop and fish farming, and livestock keeping [28]. The main crops comprise maize, cassava, sorghum and sweet potatoes. The study area has been described in detail in previous studies [29–33].

### Study design

A community based cross-sectional study was conducted in November 2017 with the objective of assessing LLNs ownership, coverage and utilization. A total of 160 households were selected from 16 villages using simple random sampling technique. A household was defined as any unit headed by a male or female with his/her dependents and/or spouse, who shared a cooking pot/common eating place and slept under the same roof [27, 34].

### Study population

The study population constituted of the inhabitants of the 16 villages in Nyabondo, Nyakach Sub-county, Kisumu County. The inclusion criteria for study participants was any adult above 18 years of age residing in the study area and willing to participate in the study. Respondents from the study population who did not consent and were unwilling to participate in the study were excluded.

### Sampling method

The study area was clustered into 16 villages based on the existing boundaries. In total there were 2, 775 households from the 16 villages, with an average of 173 households per village. A simple random sampling technique of balloting was used to select 10 households from each village (cluster) for inclusion in the study. A total of 160 households were purposefully considered given that the area has a homogeneous population that is not vast geographically. It was assumed that the population sample would constitute enough sampling frame capable of showing valid statistical difference in the study. It was estimated that the sample was adequate enough to provide valid statistical power of 80% with 95% level of precision. In each of the randomly selected household, the head or the spouse was considered for the interview.

### Data collection techniques

Data from households was collected using structured questionnaires (Supplementary file 1). Field assistants were selected from the community and trained for a day on questionnaire administration techniques. The questionnaire was developed by the researcher for this study in line with the WHO guideline and pre-tested in a non-study village. After pre-testing of the draft questionnaire, adjustments were made where necessary before the final administration. Each selected household was visited and selection procedure explained to household owners. Informed verbal consent was obtained from the household head before the start of face to face interview (Supplementary file 1). In household where the heads were absent, a spouse or an adult above 18 years was interviewed. The English version of the questionnaire was translated into the local language (Dholuo) during the interview sessions. Information collected from households inluded the frequently occurring diseases, symptoms and signs of malaria, knowledge of mosquito breeding places, LLINs ownership, use and acquisition, their condition, and the household perceptions on their use.

In total four focus group discussions (FGDs) were conducted with each one consisting of 8–10 participants (males and females aged between 30 and 60 years). The discussions were mainly conducted in English with some limited use or mixture of Kiswahili. The participants for the FGDs were members of various community groups in the study area. They group leaders assisted in selecting the participants by considering their in-depth understanding of the community perception on malaria, the vector and LLINs use in the area. The purpose of conducting FGDs was to obtain in-depth information on community perception on malaria, the vector and LLINs use. Shortly after each FGD discussion (Supplementary file 2), the facilitator and the note taker reviewed the notes for completeness and accuracy.

LLINs coverage was defined as the population level estimate of individuals who could use an LLIN based on the assumption that two people can share a net. It was calculated by multiplying the number of LLINs owned by the household by 2, creating a number of ‘potential LLIN users’ in the household. Then the number of potential LLIN users was divided by the number of household members who stayed in the house the night before the survey. Values over 1 were set to 1, as households cannot have more than 100% access. *LLINs usage* was defined as the proportion of individuals who reported using the mosquito net in the previous night [26]. Population coverage with LLINs was computed as the ratio of the total number of individuals reporting sleeping under LLINs, over the total number of individuals surveyed [25, 26].

An LLIN (long-lasting insecticidal net) was defined as a factory-treated mosquito net made with netting material that has insecticide incorporated within or bound around the fibres. The net must retain its effective biological activity without re-treatment for at least 20 WHO standard washes and 3 years of recommended use under field conditions [35]. Any net distributed through the routine free mass campaign was classified as treated as these were long-lasting insecticide-treated nets (LLINs).

With consent from the head of the household, observation and confirmation of LLINs availability and quality check were done. Spot check form was used to record the condition of each net. The condition of the net was classified as either, (i) clean/good, (ii) dirty & no holes, or (iii) dirty with holes. A net was classified as having holes if it had any finger-sized hole or larger.

### Data management and analysis

Data was recorded and entered in MS Excel, checked for errors by an independent person and processed using Statistical Package for Social Science (SPSS) version 25. Descriptive statistics were done to characterize expected outcomes like demographic household characteristics, perception of malaria and LLINs use, treatment seeking behavior, LLINs ownership, use and access. Continuous variables were expressed as mean and standard deviation (SD) while \categorical variables were expressed using frequencies and proportions. The associations between independent and dependent variables were tested using Pearson’s chi-square test. The qualitative data derived from FGDs were deductively coded and thematically analyzed by comparing common themes and responses across groups. FGDs data codes were created based on the collected qualitative data (Inductive coding).

## Results

### Socio-demographic characteristics of respondents

During the study, 65% of respondents were females and 35% were males [*n* = 160]. Around 32 % [31.9%] of the respondents had completed primary school education and 16.9% had dropped out at secondary school level. Overall, more than half [50.6%] of the respondents had either attained primary school education or dropped early during their primary education level. In terms of occupation, majority of the respondents [91.9%] were farmers, 5% in self business and 1.3% were either in brick making or not in any formal employment [Table 1]. Around 52 % (51.87%) of the households had 4–6 occupants. The average family size per household was 4.71, with a maximum of 11 and a minimum of 1 member per household.

**Table 1:** Socio-demographic characteristics of respondents

Variable	Category	Frequency (*n* = 160)	%
**Gender**	Female	104	65
	Male	56	35
**Highest education**	Primary school (Not completed)	30	18.8
	Primary school (Completed)	51	31.9
	Secondary school (Not completed)	27	16.9
	Secondary school (Completed)	25	15.6
	University/College	15	9.4
	Informal education	12	7.5
**Main occupation**	Student	1	0.6
	Farming	147	91.9
	Self-business	8	5
	Unemployed	2	1.3
	Brick making	2	1.3

### Knowledge of signs and symptoms of malaria

**Fig. 1:**
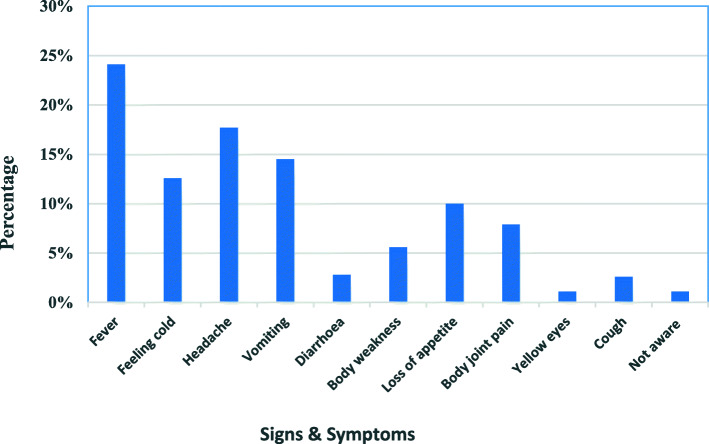
Responses regarding signs and symptoms of malaria

The commonly mentioned signs and symptoms of malaria were; fever (24.1%), headache (17.7%), vomiting (14.5%) feeling cold (12.6%) and loss of appetite (10%). Most of the malaria symptoms were mentioned across the board [Fig. 1]. The responses were scored out of 10 and placed on a 4-point likert scale where 1 indicated ‘poor’ (mentioned three and below signs/symptoms), 2 for average score (mentioned between four-five), 3 for good (between six-seven signs/symptoms), and 4 for excellent among those who mentioned above seven signs/symptoms of malaria. Based on this scale, 59.4% of the respondents could not mention more than three signs and symptoms of malaria with fever being the most commonly mentioned. Around 31 and 7.5% respondents were able to mention 4–5 and 6–7 signs and symptoms of malaria respectively. This translated to low knowledge of malaria signs and symptoms among individual household members. Common signs and symptoms of malaria were also mentioned during FGDS;

*One participants from Naki FGD stated that ‘Somebody feels body pains, cold, shivering, fever (hot), stomach ache, vomiting yellowish, joint and body pains, loss of appetite and coughs’ Similar expressions were observed in Sigoti/Nyamaroka FGDs.*

### Most frequently occurring disease in the community

In order of severity, malaria was reported as the most frequently occurring disease in the area. It was reported by 87.5% of the respondents. Besides malaria, other diseases mentioned by the respondents were typhoid [6.1%] coughs [5.6%] and flu [4.4%]. During focus group discussions, some activities and behaviors were reported to expose household members to malaria. These included, improper use of nets, getting to bed late and not tucking in nets at night. Another exposure pathway mentioned during FGDs was the common practices of community member spending considerable time outside houses in the evening without using any personal protection. Night exposure was also reported to occur during mourning and funeral ceremonies (*Disco matanga*) for dead family members where community member spend several nights outdoor without any protection against mosquito bites. These human behaviors partly explains the source of residual malaria transmission reported in the area despite high coverage and use of LLINs.

*In Naki/Sigoti FGDs participants reported that ‘During mourning at night, people don’t use nets, they don’t carry nets with them and they get exposed to mosquitoes’(‘suna’ in Dholuo language).*

### Perception of malaria risk

Children less than 5 years were said to be at higher risks of malaria infection (28.6%), followed by all children (22.2%), adult men (17.2%) and adult women (15.8%). Pregnant women and elderly people were only mentioned by (13.8%) and (2%) respectively, multiple responses were encouraged. However, 41.9% of the respondents were unable to correctly mention the group of people at highest risk of malaria infection, while 34.4 and 23.1% only managed to correctly mention one and two groups of people at risk respectively [*n* = 160]. (Table 2).

**Table 2:** Reported groups of people at higher risk of malaria infection and knowledge score on groups most affected by malaria in the community

Variable	Category	Percentage
Most affected group^a^	Adult women	15.8%
	Children under 5 years	28.6%
	All children	22.2%
	Elderly people only	2.0%
	Pregnant women	13.8%
	Adult men	17.2%
	Don’t know	0.3%
** Score**		
Score on most affected Groups	0	41.9%
	1	34.4%
	2	23.1%
	3	0.6%

^a^multiple responses were encouraged

During the FGDs, participants from all the groups mentioned children and pregnant women as the groups of people at higher risk of getting malaria in the area. Old people above 60 was also mentioned in two groups out of the four.

*‘Children between 0-5 years and pregnant women are also at risk of getting malaria (All FGDs), ‘this is because they are always tired and don’t have the strength to tuck in nets at night’*. *Old people from 60 years and above are also at higher risk. (Sigoti /Nyamaroka FGD). ‘For the old people and children, immunity is low and pregnant women are also at risk, sometimes they are tired and fail to use nets thus exposing themselves to mosquito bites’ (Siatok/Naki/Nyamaroka FGD).*

### Reported malaria incidence/ morbidity and actions taken

Around 31 % (30.6%) of the respondents reported to have had at least one member of their household sick with malaria 7 days before the interview time. Again, 8.8% of households had at least one of their members sick with the disease during the survey period. Overall, 62.5% of households reported to have experienced malaria episode 1 month prior to the study. On health seeking behavior for malaria, 72.5% of households reported to have immediately and promptly sought medical treatment (within 24 h) from a health facility or clinic first when one member of their household got malaria (Table 3). Nevertheless, 19.4 and 4.4% of the respondents reported to either buy malaria drugs from the area chemists or from the general shops once a household member got sick [*n* = 160].

**Table 3:** The last time a household member had malaria

Variable	Category	Frequency (*n* = 160)	Percentage
Last time Household member had malaria	Sick during interview	14	8.8
	1–7 Days ago	35	21.9
	1–2 Weeks ago	25	15.6
	One Month ago	26	16.3
	Two months ago	18	11.3
	Over Three months ago	20	12.5
	Can’t remember	19	11.9
	Over 1 year ago	3	1.9
Immediate action taken	No action taken	4	2.5
	Visited a HF/clinic	116	72.5
	Bought drugs from general shop	7	4.4
	Bought drugs from chemist	31	19.4
	Other actions	2	1.3

### Reported problems caused by mosquitoes

**Fig. 2:**
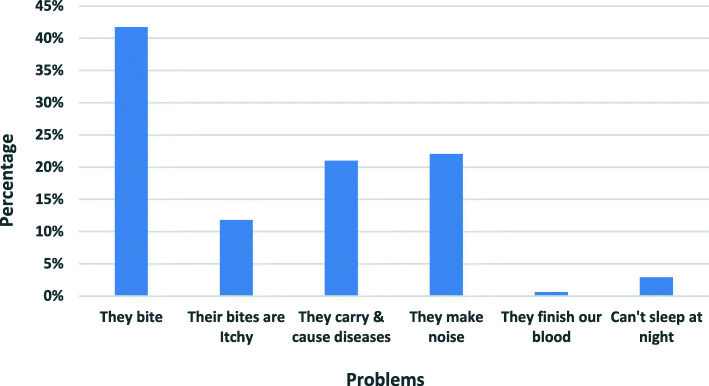
Reported problems caused by mosquitoes

Approximately 98 % (97.5%) of the respondents acknowledged that mosquitoes caused trouble in one way or another in their households. The problems mentioned were; general bites (41.70%), nuisance noise at night (22%), while disease transmission of the vector was mentioned by 21% of the respondents. Individual responses on the problems caused by the vectors were much influenced by the village of origin of the individual respondent as shown in Fig. 2 (*n* = 314). The biting nuisance of the vector was expressed during one of the FGDs, where participants in most of the groups said that sleeping late at night exposed people to mosquito bites (*Siatok /Sigoti/Nyamaroka FGD*) and use of LLINs was reported to prevent users from mosquito bites in all the groups.

### LLINs ownership, use and access

**Fig. 3:**
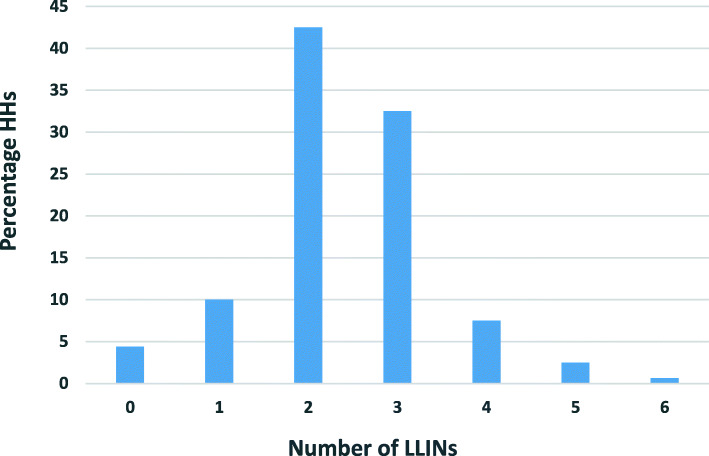
Number of LLINs owned per household

There were 382 reported LLINs among the 753 occupants of the surveyed households [*n* = 160]. The overall average of LLIN ownership per household was 2.4 (382/160) with 1.97 (753/382) persons per LLIN [computed in households that owned nets]. Around 97 % (96.9%) of households owned at least one LLIN with 64.1% owning at least one LLIN for two people. High percentage of households owned at least one LLIN, with 75% owning between 2 and 3 LLINs per household (Fig. 3).

Among those owning LLINs, 98.1% reported using them the previous night. The same percentage (98.1%) acknowledged sleeping under LLINs for the last 1 week prior to the survey. Around 4.5% of households had no nets with 32.5 and 42.5% owning 2 and 3 LLINs respectively with a mean of 2.39 and a median and mode of two each. Majority of the LLINs were owned by households with 4–6 occupants (Table 4). Based on the assumption that two people can share a net, the proportion of the population with access to an LLIN within the household was 100%. i.e. [(382*2)/753]. Additional information on LLINs ownership is also available in a case study report by the author [31].

**Table 4:** Number of LLINs owned per household occupancy size

No of LLINs	1–3 Occupants	4–6 Occupants	7–9 Occupants	Above 10 Occupants	Total HHs
0	4	1	2	0	7
1	11	3	2	0	16
2	22	38	8	0	68
3	9	29	13	1	52
4	1	8	2	1	12
5	0	3	0	1	4
6	0	1	0	0	1
Total HHs	47	83	27	3	160

### LLINs acquisition, sources and conditions

More than three quarters (76.0%) of the LLINs were acquired from free mass distribution campaigns through the Ministry of Health and 12.9% were acquired from public health facilities - given to pregnant women and mothers with children under 5 years of age visiting government owned health facilities. Eighty percent (80%) of LLINs were reported to be acquired less than 6 months with 25% being acquired less than 3 months before the survey time. On the condition of LLINs, 74.9% were in good condition with no holes while 17.2% were average despite 7.8% being damaged (with holes) (Table 5). The condition of LLINs varied significantly depending on the number of nets per household (χ2 = 44.584; *P* = 0.007) and their perceived effectiveness (χ2 = 21.358; *P* = 0.045). Retreatment of nets was not reported in the area and this was probably because conventional ITNs have been replaced by long lasting insecticide-treated nets (LLINs), requiring no retreatment.

**Table 5:** Nets acquisition and condition

		Responses (Multiple responses)	Percentage
**How nets were Acquired**			
1	From health facility	50	12.9
2	From Shop/Retail market	30	7.8
3	From mas net distribution campaign	294	76.0
4	From relative/NGOs/CBOs	13	3.4
	**Total**	387	100
**When nets were acquired**			
1	Less than 3 months ago	96	25
2	3–6 Months ago	211	55.1
3	6–12 Months ago	30	7.8
4	1–3 Years ago	42	11.0
5	3–5 Years ago	4	1.0
	**Total**	383	100
**Net Condition**			
1	Good (Clean no holes)	287	74.9
2	Average	66	17.2
3	Bad (Dirty, torn out with big holes)	30	7.8
	**Total**	383	100

### Reported benefits of using LLINs

**Fig. 4:**
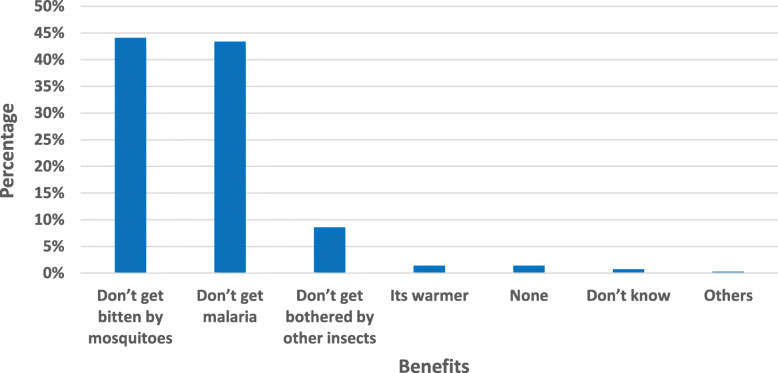
Reported benefits of sleeping under LLINs (Kindly the X-axis for Fig 4 should read 'Percentage', tried to rectify but was unable)

Two most reported benefits of LLINs use in the study area were; protection of the household members from getting bitten by indoor mosquitoes (44.1%) and protection from getting malaria (43.4%). LLINs were also reported to protect household members against bites from other nuisance insects at night (8.6%), (*n* = 290). Village of residence, number of LLINs per household, perceived effectiveness of LLINs and whether the respondent slept under net the previous night were the major variables that significantly determined each individual responses on benefits of sleeping under LLINs (Fig. 4). In general, LLINs were reported to be effective in protecting household members. Some of the above accrued benefits of using LLINs were noted during the FGD sessions. Additional information can also be found in a case study project report by Ng’ang’a et al, [31].

*‘Nets protect us from mosquito bites (all FGDs), we stay for some time without getting malaria once we use nets’ (Nyamaroka/Naki FGD). ‘Nets help us to sleep peacefully without noise disturbances from mosquitoes at night (Naki/Sigoti FGD), they also helps in reducing sickness and deaths in the community ‘hata kwa watoto’ (even among children), (Naki/Sigoti FGD).*

## Discussion

During this cross sectional household survey, malaria was reported to be one of the most frequently occurring diseases in the community, reported by 87.5% of the respondents. The government of Kenya has classified western Kenya (in the Lake Victoria region) as an epidemiological zone which is characterized by intense malaria transmission throughout the year [3, 12].

Around 63 % (62.5%) of surveyed households reported experiencing malaria episode in the previous 1 month despite reported high LLINs coverage and use. The reported residual malaria transmission in the area can be linked to vector and/or human bionomics/behaviors which compromises the contact between the vectors and the protective/control measures. These behaviors includes, mosquito feeding on humans in the absence of protection, being indoors but not under nets in the evening before going to sleep, or being outdoors away from protected houses due to occupational, domestic, recreational or cultural activities, most of which were reported during FGDs. In addition to ensuring high LLINs coverage and use in the community, it is important to educate community members on the potential risks associated with outdoor exposures and the need to minimize incidences of residual/persistent malaria transmissions.

About 73% of the respondents reported to have promptly sought treatment from a nearby health facility or clinic. The respondents’ intention to promptly seek treatment was high though slightly below the WHO Roll Back Malaria (RBM) recommendation, which states that at least 80% of those infected with malaria should seek prompt treatment within 24 h after the onset of symptoms [36]. Prompt diagnosis and effective treatment of malaria is a key component of its control and elimination. Every single case of malaria must be detected and a reliable parasitological diagnosis made. Prompt and effective treatment of malaria with appropriate drugs is expected to end the asexual cycle, and depending on the drug regimen, to kill some developing gametocytes and substantially reduce net infectiousness of individuals. It helps to prevent severe disease progression and also reduce the pool of individuals in the community who contribute to malaria transmission [37]. Several other studies from Kenya, Uganda, Ethiopia, Malawi and Ghana have reported low responses (13–37.6%) on prompt care-seeking for malaria [38–42].

Despite fever being commonly cited, there was an overall low level of knowledge on malaria signs and symptoms among household members in the area. The study findings were contrary to other related studies that have reported good knowledge among the local communities [40, 43, 44]. Improved community knowledge of malaria, its symptoms and source of transmission promotes preventive and personal protective practices amongst the affected populations [45, 46]. Knowledge of signs and symptoms of malaria also plays an important role in promoting early diagnosis and prompt treatment of malaria [45].

On LLINs ownership and use, the study showed relatively high rates of ownership and use, i.e. 96.9 and 98.1% respectively. A study conducted by Githinji et al., in 2010 [47] in nearby area of western Kenya, reported a moderate proportion of net use (59%) among household despite reported high coverage of 95% among households owning at least one net. A similar cross-sectional survey conducted in the highlands of Western Kenya [19], found that despite high ITN ownership of 71%, compliance on usage was slightly low at 56.3%. In another study in the western coastal plain of Yemen, ownership of at least one LLIN was also high (90.6%) but only 24.1% owned at least one LLINs for every two people, the overall proportion of people with access to LLINs was 51.5% and only 19.0% slept under LLINs the night before the survey [48]. Our study was consistent with another quantitative household survey conducted in China-Myanmar border in 2015 where the percentage of households that owned at least one bed net was 99.7% with 97.3% residents’ reporting to have slept under bed nets the previous night [49].

Most parts of Kenya have approached or met the RBM household LLIN coverage target of at least 60% as reported by the ministry of health [4]. One of the methods used to increase ownership in Kenya is the free distribution of LLINs to expectant mothers and children under-5 years of age through the ante-natal and post-natal clinics and mass campaigns [50]. The recorded high ownership in the study area could be attributed to the free mass-distribution campaign that took place in early 2017.

The LLINs coverage in the study area i.e. the proportion of households with at least one LLIN for every 2 household members (64.1%) was slightly below the WHO recommended level of 80% for acceptable protection [51]. The high number of LLINs ownership in the area in relation to the proportion of households with at least one LLIN for every two people indicates that nets were inequitably distributed among the households in the area (Table 5). The high LLINs ownership in the area demonstrates the pivotal role mass-distribution campaigns plays in ensuring that residents in endemic malaria areas own LLINs. The average family size per household in the study area was 4.71, with a maximum of 11 and a minimum of 1 member per household. The mean bednet density was approximately 2.4 bednets per household and on average 1.97 persons per bednet, which is almost two household members per net as per WHO target [25, 26, 51, 52]. LLINs use at household level varied significantly based on; village of origin, education level of respondent and respondents’ knowledge of personal protection methods. Other studies have also shown association between knowledge on bednets and bednet ownership. For example, in the Cross River state of Nigeria [53], it was reported that educated parents were able to appreciate the importance of treated nets in malaria prevention and eventually influenced bednet usage. Such associations have also been reported in Kinshasa-DRC [54, 55] and in Mfou-Cameroon [56].

On LLINs acquisition, more than three quarters (76.0%) of the nets were acquired from free mass distribution campaigns through the Kenyan Government, Ministry of Health. LLIN distribution through mass campaigns offers opportunities to rapidly increase LLIN coverage in targeted communities (“catch-up”) but is most effective when implemented in parallel with continuous distribution through routine antenatal or immunization services to maintain coverage (“keep-up”) [3, 10–12, 50]. These distribution programmes can rapidly increase ownership and bolster household use. For example, in Sierra Leone, a mass distribution campaign increased household use by 137% within 6 months [57].

Spot-checks on the physical condition of LLINs during our survey revealed that 74.9% of them were in good condition and 7.8% were damaged with holes. Most of the nets were in good condition and this was because majority of them (80.2%) were acquired less than 6 months prior to the survey. Our finding was slightly different from a related study conducted in Kenya by Githinji et al.*,* 2010, [47], who reported poor physical condition (40%) of the nets. And in another related study in Burundi, despite high rate of LLINs distribution among targeted households, their lifespan and fabric quality was reported to decrease quickly after developing holes [58]. Likewise, a net survey in Tanzania revealed that 44.9% of the nets had holes [59]. Major observed causes of holes in LLINs is due to the commonly used wooden beds and the sticks used to support the nets around the sleeping areas.

The two most reported benefits of using LLINs in the study area were; protection of the household members from getting bitten by indoor mosquitoes (44.1%) and protection from getting malaria (43.4%). In order to sustain nets use in the community, it is important to find out what motivates people to use nets and what prevents them from using them. For example, preventing nuisance biting is often a much stronger motivator for bed nets ownership and use than preventing malaria. This tendency has been reported in studies conducted in Zanzibar and Kenya [17, 60]. However an important limitation of this type of motivation is that household members might end up only using bed nets when the mosquito density is high. It might also create the impression that bed nets are simple luxury items and not a priority for malaria control in endemic areas [61]. In a study in Ghana, households used ITNs to reduce the nuisance caused by mosquitoes, and not to prevent malaria [62]. However, in western Kenyan highlands, another study showed that seasonal patterns of precipitation and vector density, along with education, were associated with ITN use [19]. The non-use of nets may also be related or influenced by other factors such as social status [18], cost, access, socio-economic factors, perceived benefits, effectiveness, demographic disparities, culture, ethnicity, education, gender, shape of the net, sleeping arrangements [17, 63, 64], household characteristics [20, 65], condition of the nets, colour, and gender among others [17, 23].

Another motivating factor for LLINs use is their potential to offer protection against other vector borne diseases such as leishmaniasis, Japanese encephalitis, dengue and lymphatic filariasis [66]. Elsewhere LLINs have been reported to not only provide protection against nuisance mosquitoes, but to also kill head lice and bedbugs, which contributes greatly to their acceptance and use by some communities [66, 67].

### Study limitations

One of the limitations of the study was that information on LLINs use the previous night was obtained by self-reporting, without being confirmed through visual inspection at night. The condition of the nets was assessed using a checklist/form that might not be the WHO standard method of using proportional whole index of measuring net condition. However, despite these limitations, this study presents findings that are relevant for sustaining universal coverage and utilization of LLINs in the study area as well as in other malaria endemic areas of Sub Saharan Africa.

## Conclusion

The level of LLIN coverage, access, ownership and use was high in the study area, almost attaining the national targets of 100% for ownership and above 80% for use. However, majority of households (62.5%) reported experiencing malaria episode in the previous 1 month in the study area. There is need to monitor and determine the fraction of the population fully protected by LLINs while at the same time assessing the likelihood of malaria transmission outdoors where protection with LLINs is not feasible.

## Supplementary Information

**Additional file 1.** LLINs use household questionnaire.

**Additional file 2.** FGD interviewers guide.

## Data Availability

The original data and materials used for this study are available from the corresponding author for non-commercial purposes upon request.

## Abbreviations

IRS: Indoor residual spraying
LLINs: Long-lasting insecticidal nets
FGD: Focus Group Discussion
ITNs: Insecticide-treated Nets
KEMRI: Kenya Medical Research Institute
RBM: Roll Back Malaria Partnership
SERU: Scientific and Ethic Review Unit
VBD: Vector-Borne Diseases
WHO: World Health Organization.
IVM: Integrated Vector Management

## References

[1] World Health Organization (2020). World Malaria Report.

[2] World Health Organization (2019). World Malaria Report.

[3] National Malaria Control Programme [NMCP]. Ministry of Health. The epidemiology and control profile of malaria in Kenya: reviewing the evidence to guide the future vector control. Nairobi: National Malaria Control Programme, Ministry of Health; 2016.

[4] DOMC. Towards a malaria-free Kenya. National Malaria Strategy 2009–2017. Division of malaria control. Nairobi: 2019 Ministry of Public Health and Sanitation; 2009.

[5] Bhatt S, Weiss DJ, Cameron E, Bisanzio D, Mappin B, Dalrymple U, Battle KE, Moyes CL, Henry A, Eckhoff PA, Wenger EA, Briet O, Penny MA, Smith TA, Bennett A (2015). The effect of malaria control on *Plasmodium falciparum* in Africa between 2000 and 2015. Nature.

[6] Lengeler C (2004). Insecticide-treated nets for malaria control: real gains. Bull World Health Organ.

[7] World Health Organisation (2016). World Malaria Report.

[8] World Health Organization (2020). WHO malaria fact sheet.

[9] Guyatt HL, Corlett SK, Robinson TP, Ochola SA, Snow RW (2002). Malaria prevention in highland Kenya: indoor residual house-spraying vs. insecticide-treated bednets. Tropical Med Int Health.

[10] Zhou G, Li JS, Ototo EN, Atieli HE, Githeko AK, Yan G (2014). Evaluation of universal coverage of insecticide-treated nets in western Kenya: field surveys. Malar J.

[11] Zhou G, Lee M-C, Githeko AK, Atieli HE, Yan G (2016). Insecticide-treated net campaign and malaria transmission in Western Kenya: 2003–2015. Front Public Health.

[12] National Malaria Control Programme [NMCP] (2019). Kenya Malaria Strategy 2019–2023.

[13] Noor AM, Amin AA, Akhwale WS, Snow RW (2007). Increasing coverage and decreasing inequity in insecticide-treated bed net use among rural Kenyan children. PLoS Med.

[14] Chuma J, Okungu V, Ntwiga J, Molyneux C (2010). Towards achieving Abuja targets: identifying and addressing barriers to access and use of insecticides treated nets among the poorest populations in Kenya. BMC Public Health.

[15] Matovu F, Goodman C, Wiseman V, Mwengee W (2009). How equitable is bed net ownership and utilisation in Tanzania? A practical application of the principles of horizontal and vertical equity. Malar J.

[16] Wanzira H, Yeka A, Kigozi R, Rubahika D, Nasr S, Sserwanga A, Kamya M, Filler S, Dorsey G, Steinhardt L (2014). Longlasting insecticide-treated bed net ownership and use among children under five years of age following a targeted distribution in Central Uganda. Malar J.

[17] Ng’ang’a P, Jayasinghe G, Kimani V, Shililu J, Kabutha C, Kabuage L, Githure J, Mutero C (2009). Bed net use and associated factors in a rice farming community in Central Kenya. Malar J.

[18] Ernst KC, Hayden HH, Olsen H, Cavanaugh JL, Ruberto I, Agawo M, Munga S (2016). Comparing ownership and use of bed nets at two sites with differential malaria transmission in western Kenya. Malar J.

[19] Atieli HE, Zhou G, Afrane Y, Lee MC, Mwanzo I, Githeko AK, Yan G (2011). Insecticide-treated net (ITN) ownership, usage, and malaria transmission in the highlands of western Kenya. Parasit Vectors.

[20] Iwashita H, Dida G, Futami K, Sonye G, Kaneko S, Horio M, Kawada H, Maekawa Y, Aoki Y, Minakawa N (2010). Sleeping arrangement and house structure affect bed net use in villages along Lake Victoria. Malar J.

[21] Macintyre K, Littrell M, Keating J, Hamainza B, Miller J, Eisele TP (2012). Determinants of hanging and use of ITNs in the context of near universal coverage in Zambia. Health Policy Plan.

[22] Hetzel MW, Gideon G, Lote N, Makita L, Siba PM, Mueller I (2012). Ownership and usage of mosquito nets after four years of large-scale free distribution in Papua New Guinea. Malar J.

[23] Fokam EB, Kindzeka GF, Ngimuh L, Kevin TJ, Dzi KTJ, Wanji S (2017). Determination of the predictive factors of long-lasting insecticide-treated net ownership and utilisation in the Bamenda Health District of Cameroon. BMC Public Health.

[24] Storey JD, Babalola SO, Ricotta EE, Fox KA, Toso M, Lewicky N, Koenker H (2018). Associations between ideational variables and bed net use in Madagascar, Mali, and Nigeria. BMC Public Health.

[25] Roll Back Malaria (RBM) (2013). Monitoring and Evaluation Reference Group Survey and Indicator Task Force. Household survey indicators for malaria control.

[26] Koenker H, Arnold F, Ba F, Cisse M, Diouf L, Eckert E, Erskine M, Florey L, Fotheringham M, Gerberg L, Lengeler C, Lynch M, Mnzava A, Nasr S, Ndiop M, Poyer S, Renshaw M, Shargie E, Taylor C, Thwing J, van Hulle S, Ye Y, Yukich J, Kilian A (2018). Assessing whether universal coverage with insecticide-treated nets has been achieved: is the right indicator being used?. Malar J.

[27] KNBS. Kenya Population and Housing Census: Volume I (2019). Population by County and Sub-County.

[28] Ng’ang’a PN, Mutunga J, Oliech G, Mutero CM (2019). Community knowledge and perceptions on malaria prevention and house screening in Nyabondo, Western Kenya. BMC Public Health.

[29] Imbahale SS, Abonyo OK, Aduogo OP, Githure JI, Mukabana WR (2013). Conflict between the need for income and the necessity of controlling endemic malaria. J Ecosyst Ecography.

[30] Mutero C, Mbogo C, Mwangangi J, Imbahale S, Kibe L, Orindi B, Girma M, Njui A, Lwande W, Affognon H, Gichuki C, Mukabana W (2015). An Assessment of Participatory Integrated Vector Management for Malaria Control in Kenya. Environ Health Perspect.

[31] Ng’ang’a PN, Aduogo P, Mutero CM (2021). Strengthening community and stakeholder participation in the implementation of integrated vector management for malaria control in western Kenya: a case study. Malar J.

[32] Imbahale SS, Paaijmans KP, Mukabana WR, Lammeren R, Githeko AK, Takken W (2011). A Longitudinal study on Anopheles mosquito larval abundance in distinct geographical and environmental settings in western Kenya. Malar J.

[33] Ng'ang'a PN, Okoyo CO, Mbogo C, Mutero CM (2020). Evaluating effectiveness of screening house eaves as a potential intervention for reducing indoor vector densities and malaria prevalence in Nyabondo, western Kenya. Malar J.

[34] KNBS (2018). Kenya population and housing census.

[35] World Health Organization (2010). Insecticide-treated mosquito nets: a WHO position statement.

[36] World Health Organization (2008). World Malaria Report.

[37] World Health Organization (2015). Guidelines for the treatment of malaria.

[38] Chuma J, Abuya T, Memusi D, Juma E, Akhwale W, Ntwiga J, Nyandigisi A, Tetteh G, Shretta R, Amin A (2009). Reviewing the literature on access to prompt and effective malaria treatment in Kenya: implications for meeting the Abuja targets. Malar J.

[39] Nuwaha F (2002). People’s perception of malaria in Mbarara, Uganda. Tropical Med Int Health.

[40] Deressa W, Ali A, Enquoselassie F (2003). Knowledge, attitudes and practices about malaria, the mosquito and antimalarial drugs in a rural community. Ethiop J Health Dev.

[41] Kabaghe (2018). Access and adequate utilization of malaria control interventions in rural Malawi: a descriptive quantitative study. Malar J.

[42] Dunyo SK, Afari EA, Koram KA, Ahorlu CK, Abubakar I, Nkrumah FK (2000). Health Centre versus home presumptive diagnosis of malaria in southern Ghana: implications for home-based care policy. Trans R Soc Trop Med Hyg.

[43] Chovatiya SK, Gajera NB, Soni VC (2013). People's perception on malaria: a case study in rural areas of Rajkot district, Gujarat-India. Health Sci Int J.

[44] Joshi AB, Banjara MR (2008). Malaria related knowledge, practices and behaviour of people in Nepal. J Vector Borne Dis.

[45] Dlamini SV, Liao WW, Dlamini ZH, Siphepho JS, Cheng PC, Chuang TW, Fan CK (2017). Knowledge of human social and behavioral factors essential for the success of community malaria control intervention programs: The case of Lomahasha in Swaziland. J Microbiol Immunol Infect.

[46] Ahorlu CK, Koram KA, Arholu C, De Savigny D, Weiss MG (2006). Socio-cultural determinants of treatment delay for childhood malaria in southern Ghana. Tropical Med Int Health.

[47] Githinji S, Herbst S, Kistemann T, Noor AM (2010). Mosquito nets in a rural area of western Kenya: ownership, use and quality. Malar J.

[48] Al-Eryani SMA, Mahdy MAK, Al-Mekhlafi AM (2017). Access to and use of long-lasting insecticidal nets and factors associated with non-use among communities in malaria-endemic areas of Al Hudaydah governorate in the Tihama region, west of Yemen. Malar J.

[49] Liu H, Xu J, Guo X, Havumaki J, Lin Y, Yu G, Zhou D (2015). Coverage, use and maintenance of bed nets and related influence factors in Kachin special region II, northeastern Myanmar. Malar J.

[50] DOMC (2011). Kenya Malaria Indicator Survey.

[51] World Health Organisation (2014). Recommendations for Achieving Universal Coverage with Long-Lasting Insecticidal Nets in Malaria Control.

[52] Koenker H, Kilian A (2014). Recalculating the net use gap: a multi-country comparison of ITN use versus ITN access. PLoS One.

[53] Eteng M, Mitchell S, Garba L, Onebieni A, Liman M, Cockcroft A, Neil A (2014). Socio-economic determinants of ownership and use of treated bednets in Nigeria: results from a cross-sectional study in Cross River and Bauchi states in 2011. Malar J.

[54] Pettifor A, Taylor E, Nku D, Duvall S, Tabala M, Mwandagalirwa K, Meshnick S, Behets F (2009). Free distribution of insecticide treated nets to pregnant women in Kinshasa: an effective way to achieve 80% use by women and their newborns. Tropical Med Int Health.

[55] Ndjinga JK, Minakawa N (2010). The importance of education to increase the use of bed nets in villages outside of Kinshasa, Democratic Republic of the Congo. Malar J.

[56] Tchinda VHM, Socpa A, Keundo AA, Zeukeng F, Seumen CT, Leke RG, Moyou RS (2012). Factors associated to bednet use in Cameroon: retrospective study in Mfou health district in the Centre region. Pan Afr Med J.

[57] Bennett A, Smith SJ, Yambasu S, Jambai A, Alemu W, Kabano A, Eisele TP (2012). Household possession and use of insecticide-treated mosquito nets in Sierra Leone 6 months after a national mass-distribution campaign. PLoS One.

[58] Protopopoff N, Van Bortel W, Marcotty T, Van Herp M, Maes P, Baza D, D’Alessandro U, Coosemans M (2007). Spatial targeted vector control in the highlands of Burundi and its impact on malaria transmission. Malar J.

[59] Maxwell CA, Rwegoshora RT, Magesa SM, Curtis CF (2006). Comparison of coverage with insecticide-treated nets in a Tanzanian town and villages where nets and insecticide are either marketed or provided free of charge. Malar J.

[60] Beer N, Ali AS, Eskilsson H, Jansson A, Abdul-Kadir FM (2012). A qualitative study on caretakers' perceived need of bed-nets after reduced malaria transmission in Zanzibar, Tanzania. Public Health.

[61] Kroeger A, Mancheno M, Alarcon J, Pesse K (1995). Insecticide-impregnated bednets for malaria control: varying experiences from Ecuador, Colombia and Peru concerning acceptability and effectiveness. Am J Trop Med Hyg.

[62] Adongo P, Kirkwood B, Kendall C (2005). How local community knowledge about malaria affects insecticide treated net use in northern Ghana MIM-PA-238950. Acta Trop.

[63] Kanmiki EW, Awoonor-Williams JK, Phillips JF, Kachur SP, Achana SF, Akazili J, et al. Socio-economic and demographic disparities in ownership and use of insecticidetreated bed nets for preventing malaria among rural reproductive-aged women in northern Ghana. PLoS One. 2019;14(1):1–13. 10.1371/journal.pone.0211365 PMC6350974 30695044

[64] Malede A, Aemero M, Gari SR, Kloos H, Alemu K (2019). Barriers of persistent long-lasting insecticidal nets utilization in villages around Lake Tana, Northwest Ethiopia: a qualitative study. BMC Public Health.

[65] Plucinski MM, Chicuecue S, Macete E, Chambe GA, Muguande O, Matsinhe G, Colborn J, Yoon SS, Doyle TJ, Kachur SP, Aide P, Alonso PL, Guinovart C, Morgan J (2015). Sleeping arrangements and mass distribution of bed nets in six districts in central and northern Mozambique. Tropical Med Int Health.

[66] Stanton MC, Bockarie MJ, Kelly-Hope LA (2013). Geographical factors affecting bed net ownership, a tool for the elimination of Anopheles-transmitted lymphatic Filariasis in hard-to-reach communities. PLoS One.

[67] Wilson AL, Dhiman RC, Kitron U, Scott TW, van den Berg H, Lindsay SW (2014). Benefit of insecticide-treated nets, curtains and screening on vector borne diseases, excluding malaria: a systematic review and meta-analysis. PLoS Negl Trop Dis.

