# Supplementary material for: Long lasting insecticidal mosquito nets (LLINs) ownership, use and coverage following mass distribution campaign in Lake Victoria basin, Western Kenya
Source: BMC Public Health. 2021 Jun 2;21:1046. doi: 10.1186/s12889-021-11062-7 (PMC8173981; doi:10.1186/s12889-021-11062-7)
Supplement: Supplementary file 1 — Additional file 1. LLINs use household questionnaire. [file 12889_2021_11062_MOESM1_ESM.pdf]

HOUSEHOLD QUESTIONNAIRE ON LLINs USE –NYABONDO KENYA

INTRODUCTION

"Hello, my name is ………………..from ICIPE. I/we are carrying out a survey on LLINs use in this area. I hope you can spare some of your time by helping us answer some few questions. Your participation will enable us to better understand the malaria situation and LLINs use in your community. I hope that you will feel free to respond to few questions and other issues that involve your household. You are not under obligation to participate in the study, but it is my desire that you do so. All information received from you will be strictly confidential and will be stored and used safely for the intended purpose. Do you agree?

Yes______________ No______________

(If yes, thank the respondent and proceed to the rest of the questionnaire).

NB: In the event that information is not made available after repeated visits please fill the box below and return the questionnaire.

**SECTION 1 : IDENTIFICATION**		
1	Date of interview/visit	
2	Village name/code	
3	Household number (if list provided)	
4	Name of interviewer	
5	Name of respondent	
6	Gender of Respondent	
7	Number of occupants	
8	Number of Nets	

9. Highest level of education attained

1	Primary school (Not completed)	
2	Primary school (Completed)	
3	Secondary school (Not completed)	
4	Secondary school (Completed)	
5	University/ college	
6	Informal	

10. Main Occupation

1	Student	
2	Farming	
3	Self business	
4	Salaried Employment	
5	Unemployed	
6	Others (Specify)	

11. What are the three most frequently occurring diseases in your household? –in order of severity

1. -----------------------------
2. -----------------------------
3. ------------------------------

12. When was the last time a member in your household had malaria?

	**When**	**Tick**
1	A weeks ago	
	Two weeks ago	
2	One month ago	
3	Two months ago	
4	Over Three months ago	
5	Can’t remember	
6	Any Other (Specify)	

13. What immediate action did you take when one of your family member suffered malaria

	**Action Taken** **(Within 24Hrs)**	**Tick**
1	Visited a local HF/Clinic	
2	Visited a traditional healer	
3	Bought drugs from general shop	
4	Bought drugs from chemist	
5	Took traditional herbs	
6	Other (Specify)	
7		

14. What are the symptoms and signs of malaria you are aware of? (*Multiple answers possible*)

	**Symptoms/ signs**	**Tick**
1	Fever	
2	Feeling cold	
3	Headache	
4	Vomiting	
5	Diarrhoea	
6	General body weakness	
7	Loss of appetite	
8	Body pain/joint pain	

9	Convulsion	
10	Eyes become yellow	
11	Cough	
12	Don’t know	
13	Others (Specify)	

16. Which categories /groups of people do you think are most affected by malaria? (*Prompt; multiple answers)*

	**Category**	**Tick**
1	Adults women	
2	Children under five years	
3	All children	
4	Elderly people only	
5	Pregnant women	
6	Adult men	
7	Don’t know	
8	Others (Specify)	

17. Do mosquitoes cause any trouble to you or your family?

1. Yes □ 2.No □ (If No go to Qn. 14)

18. In what ways do they cause you trouble? (Do not prompt)

	**Cause you trouble**	**Tick**
1	They bite	
2	Their bites are itchy	
3	They carry and cause diseases	
4	They make noise	
5	They finish our blood	
6	Can’t sleep at night	
7	Others (specify)	

19. Are you aware of any personal protection method(s) of controlling or protecting you and your family from malaria/mosquito bite?

1	Yes
2	No

20. If Yes, what are some of the ways of protecting your household/family from malaria/Mosquito bite? Indicate how often you apply each methods and rank them in order of effectiveness (*Tick accordingly as they are mentioned*).

Codes: **Application:** **1**.Daily **2.**Weekly **3.** Fortnightly **4.** Monthly **5**. More than monthly

Effectiveness: **1.** Effective **2.** Not so effective **3.** Not effective at all

	**Method**	**Methods Known to you** *(tick)*	**Methods Currently Applying** *(tick)*	**Application frequency** *(Prompt)*	**Effectiveness** *(Prompt)*
1	Use of mosquito net-untreated				
2	Use of treated mosquito net [LLNs]				
3	Use of insecticide spray				
4	Taking preventive medicine				
5	Screen windows and doors				
6	Light the fire/coils				
7	Apply mosquito repellents to the skin				
8	Use traditional Methods (Specify)				
9	Others (Specify)				

21. Do you have a Bed net [LLNs] in your household? (Should be asked only if Bednets have not been mentioned before)

1.	Yes		NO	

22. How were your nets acquired, their colour and current condition

	**How acquired (Code)**	Colour	When acquired	Current Condition
1				
2				
3				
4				
5				
6				
7				

How acquired : 1).From a health facility **2)**. From a Shop/Retail market **3)**. From mass distributiom campaign **4).** Fom a relative/NGO/CBO

When acquired : **1).** Less than 3 months ago **2)**. Between 3-6 months ago **3)**. Between 6-12 months ago -----------------------**4).**More than one year ago

Current condition : 1). Good (clean & no holes) **2).** Average **3)** .Bad (Dirty, torn out with holes)

23. Did you sleep under LLNs last night

1.	Yes		**2**	NO		3	No Net	

24. In the last one week, how often have you slept under insceticide treted net

	How often	Tick
1	Only Once	
2	Regularly-More than once	
3	Always	
4	Never	
5	No Net	

25. What do you think are the benefits of sleeping under a bednet? (*Multiple answers, don't prompt)*

	Reasons	Tick
1	Don't get bitten by Mosquitoes	
2	Don't get malaria	
3	Don't get bothered by other insects	
4	Its warmer	
5	None	
6	Don't know	
7	Others (Specify)	

-------- END --------

(Thank the respondent for sparing his/her time to participate).
