# Supplementary material for: Long lasting insecticidal mosquito nets (LLINs) ownership, use and coverage following mass distribution campaign in Lake Victoria basin, Western Kenya
Source: BMC Public Health. 2021 Jun 2;21:1046. doi: 10.1186/s12889-021-11062-7 (PMC8173981; doi:10.1186/s12889-021-11062-7)
Supplement: Supplementary file 2 — Additional file 2. FGD interviewers guide. [file 12889_2021_11062_MOESM2_ESM.pdf]

LLINs FOCUS GROUP DISCUSSIONS GUIDELINE

Introduction

"Hello, my name is ………………….from ICIPE. I/we are carrying out a study on malaria control and LLINs use in your community. I hope you can help us by participating in some focused discussions on selected topics. Your participation will enable us to better understand the malaria situation, LLINs use and coverage status in this area. I hope that you will feel free to participate and discuss with us. You are not under obligation to participate in the discussion, but it is my desire that you do so.

Questions for discussion

1. (a) Is malaria a major health problem in this area? Why
   (b) What activities expose people to mosquito bites and malaria in this area?

2. (a) What are the **most common** symptoms associated with malaria in this area and how do local people differentiate malaria symptoms with those of other illnesses?

   (b) How do **malaria symptoms** present/manifest in a patient?

3. Which categories /groups of people are most affected (at higher risk) by malaria in this area/village? And what makes this categories to be at a high risk ?

4. (a) Which are the applied personal protection methods for malaria in this area?
   (b) What are peoples perception on their effectiveness?

5. Do people always sleep under insceticide treted net in this area? If Not, Why?
6. What are peoples perception on LINNs effectiveness?

7. How are LLINs owned in this area?

8. If a net is acquired in the household, who will be given priority to use?

9. When are bednets mostly used time (eg. Time of the days/days/ seasons/years)

10. What do local people think are the benefits of sleeping under LLINs ?

End of the discussion.

Thank all the participants for their contribution and spending their precious time with you.
